# A phase 2 study of stereotactic body radiation therapy for squamous cell carcinoma of the head and neck (SHINE): a single arm clinical trial protocol

**DOI:** 10.1186/s12885-023-10807-4

**Published:** 2023-04-26

**Authors:** Justin Lee, Nhu Tram Nguyen, James Wright, Ka-Kit David Yeung, Stephen Sagar, Do-Hoon Kim, Orest Ostapiak, Lilian Doerwald-Munoz, Timothy Whelan

**Affiliations:** 1 Department of Oncology, Faculty of Health Sciences, McMaster University, Hamilton, Canada; 2 Juravinski Cancer Centre, Hamilton Health Sciences, 699 Concession Street, Hamilton, ON, CA L8V 5C2 Canada

**Keywords:** Stereotactic radiation therapy, Head and neck cancer, Squamous cell carcinoma, Radiation therapy

## Abstract

**Background:**

Cancers of the head and neck region are often characterized by locally advanced, non-metastatic disease. Standard treatments for advanced cervico-facial cancers of the skin or primary head and neck squamous cell carcinoma (HNSCC) include combinations of surgery, radiation and chemotherapy, which are associated with high rates of acute toxicity and complications. Stereotactic body radiotherapy (SBRT) has been shown to be a promising modality of treatment for this patient population in retrospective studies; to our knowledge, there are no prospective clinical studies evaluating the safety and efficacy of SBRT in these patients.

**Methods:**

This phase 2, single institution, single arm study aims to evaluate response rates to SBRT in older age patients with locally advanced HNSCC for whom primary surgery is not recommended or performed. The intervention is SBRT 45 Gy in 5 fractions given every 3–4 days. Toxicity, quality of life and patient outcomes will be recorded regularly up to 24 months after completion of SBRT.

**Discussion:**

For this patient population, SBRT may offer a shorter and more effective treatment than the current standard of care palliative regimens. If the study demonstrates that SBRT is safe and effective, then this may lead to randomized studies comparing conventional radiotherapy to SBRT for selected head and neck cancer patients.

**Trial registration:**

ClinicalTrials.gov Identifier: NCT04435938.  Date registered: June 17, 2020.

**Supplementary Information:**

The online version contains supplementary material available at 10.1186/s12885-023-10807-4.

## Background

Cancers of the head and neck region account for approximately 4% of all new cancer cases. Primary skin cancers are the most common malignancy diagnosed in North America with the majority of tumours arising in the cervico-facial region [1, 2]. Together, these tumours comprise a high burden of illness and are often characterized by locally advanced, non-metastatic disease.

Determining the optimal treatment for individual patients with advanced cervico-facial cancers of the skin or primary head and neck squamous cell carcinoma (HNSCC) is clinically challenging; standard treatments include combinations of surgery, radiation and chemotherapy, all of which are associated with high rates of acute toxicity and complications [3]. A meta-analysis of randomized controlled trials did not demonstrate benefit with concurrent chemotherapy in patients over the age of 70 or with performance status ≥ 2, and it is recognized that the high burden of medical co-morbidities in HNSCC is associated with poorer prognosis [4-6]. Some patients without distant metastases may be deemed to have ‘incurable’ disease due to very advanced tumours, recurrence, severe medical co-morbidities or frailty that prohibit the use of standard surgery, general anaesthetic and/or radiotherapy over 6–7 weeks [7-9].

When conventional surgery and/or radiotherapy are not recommended by the multi-disciplinary team then patients may be treated with shorter, hypo-fractionated radiotherapy with the goal of symptom relief and local control but at the cost of a lower biological dose. Investigators at the Juravinski Cancer Centre published retrospective results from the ‘0–7-21’ regimen using 24 Gy / 3 fractions which was well tolerated and provided temporary symptom relief in 82% of patients but reported 6 month progression free survival of 39% within the irradiated field [7]; a phase 2 study of previously untreated HNSCC patients deemed to have incurable disease used up to 42 Gy/12 fractions and demonstrated similar rates of initial response and symptom relief but a short progression free survival duration of 3.1 months [8]. Stevens et al. reported an institutional experience of palliative radiotherapy in newly diagnosed head and neck cancer patients who were deemed to have incurable disease and received a wide range of dose/fractionation regimens. The median radiation dose was 50 Gy and between 57–82% of patients were reported to have any radiological, clinical or symptomatic response to treatment [9]. In these three studies, the patients were older with median ages of 71, 73, and 77 years- and median survival was short 5.2, 5.7 and 6.2 months [7-9].

With respect to squamous cell carcinoma (SCC) of the skin, there is limited evidence to guide treatment in patients with unresectable or medically inoperable disease, particularly in the head and neck region [10]. There is a need for prospective data on non-surgical treatment options for frail older adults which improve efficacy while limiting the treatment burden [11].

SBRT can limit the number of treatments while delivering a higher, potentially curative dose. An international consortium of 15 high volume cancer centres reported on a survey of practices using SBRT for head and neck cancers [12]. There was heterogeneity in the indications, techniques and doses reported by various institutions. The most common indication was in the setting of recurrent disease and reported doses were in the range of 35–50 Gy in 3–5 fractions. Several institutions reported 1–2 year local control rates of 65–90% with SBRT [12-15] and acceptable levels of toxicity. To our knowledge, there are no prospective clinical studies evaluating tumour response, toxicity and quality of life in previously unirradiated patients.

The goal of the current study is to prospectively evaluate tumour response, toxicity and patient quality of life in patients with HNSCC undergoing SBRT.

## Study objectives

### Primary objective

To evaluate the tumour response rate of squamous cell carcinoma of the head and neck following stereotactic body radiotherapy (SBRT).

### Secondary objectives:

1. To measure acute and late toxicity rates associated with SBRT treatments to the head and neck region
2. To measure patient reported outcomes including quality of life and specific symptoms associated with head and neck cancer and SBRT treatment
3. To determine local control, locoregional control and progression free survival following SBRT

## Study design

A phase 2, single arm study to evaluate response rate to SBRT in patients with locally advanced squamous cell carcinoma of the head and neck region, including primary skin SCC (Scheme I).

**Scheme I:**
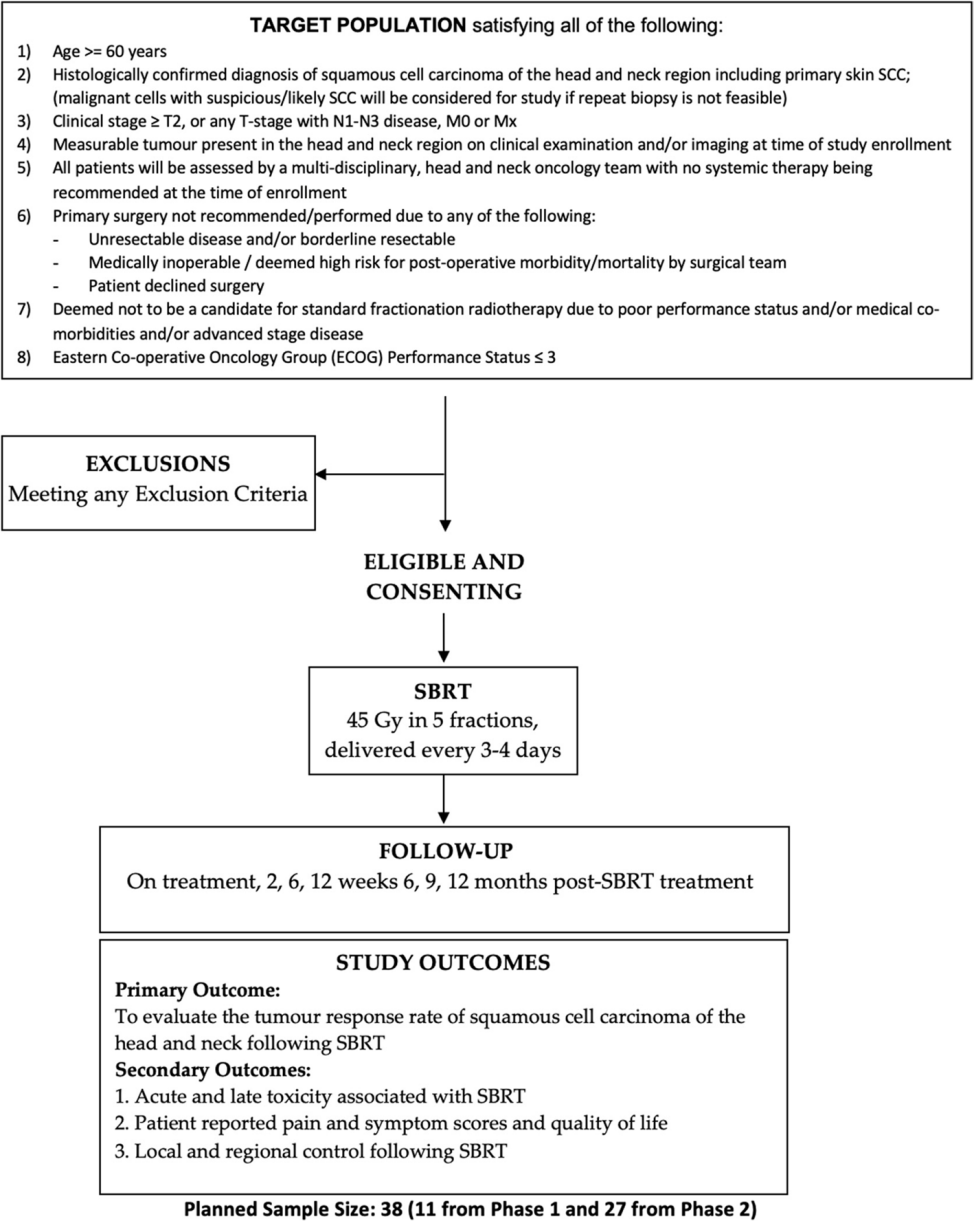
Study Schema**.** Abbreviations: SCC = squamous cell carcinoma; SBRT = stereotactic body radiotherapy

## Study population

### Inclusion criteria.

1. Age >  = 60 years
2. Histologically confirmed diagnosis of squamous cell carcinoma of the head and neck region including primary skin SCC; (malignant cells with suspicious/likely SCC will be considered for study if repeat biopsy is not feasible)
3. Clinical stage ≥ T2, or any T-stage with N1-N3 disease, M0 or Mx
4. Measurable tumour present in the head and neck region on clinical examination and/or imaging at time of study enrollment
5. All patients will be assessed by a multi-disciplinary, head and neck oncology team with no systemic therapy being recommended at the time of enrollment
6. Primary surgery not recommended/performed due to any of the following:
  Unresectable disease and/or borderline resectable
  Medically inoperable / deemed high risk for post-operative morbidity/mortality by surgical team
  Patient declined surgery
7. Deemed not to be a candidate for standard fractionation radiotherapy due to poor performance status and/or medical co-morbidities and/or advanced stage disease
8. Eastern Co-operative Oncology Group (ECOG) Performance Status ≤ 3

### Exclusion criteria.

1. Life expectancy ≤ 3 months
2. Chemotherapy or other systemic cancer therapy within 3 months prior to HN SBRT
3. Basal cell carcinoma, Merkel cell, malignant melanoma, adenocarcinoma are excluded
4. HN surgery within 6 months prior to HN SBRT (excision under local anaesthesia is acceptable)
5. Prior radiation treatment to the head and neck region (prior radiotherapy to the skin for non-melanoma skin cancer and deemed to have no risk of overlap with the current field are eligible)
6. Synchronous or recent cancer diagnosis not including the index cancer (other cancers treated curatively with no evidence of disease for >  = 3 years, or other non-melanoma skin cancers treated with no evidence of disease for >  = 6 months are eligible)
7. Confirmed or known distant metastatic disease
8. Serious non-malignant disease that precludes definitive radiation treatment (e.g. severe cases of scleroderma, systemic lupus erythematosus, rheumatoid arthritis)
9. Unable to provide written, informed consent or complete QoL questionnaires and assessments required on the study
10. Unable to lie flat for 60 min in order to have radiation planning and treatment
11. Unable to attend radiation planning and therapy, as well as follow-up care and assessments
12. Unable to provide written, informed consent

## Pretreatment evaluation and subject enrollment

Following the multi-disciplinary consultation including a head and neck surgeon and radiation oncologist, patients who meet the inclusion criteria will be screened and approached for eligibility assessment and potential study enrollment. Written informed consent is obtained for all eligible patients prior to commencing any study related procedures.

All potentially eligible and consenting patients who do not meet the exclusion criteria will have a CT simulation of head and neck region. Based on our experience in the head and neck clinic, less than 10% of patients in this population will be unable to tolerate the CT with head and neck immobilization. Patients who cannot tolerate the simulation procedure with immobilization will be treated off study.

## Baseline assessment

1. Complete history and physical examination; multi-disciplinary clinic consult
2. The baseline assessment includes the collection of patient demographics, height, weight, tumour characteristics (disease site, histolology, TNM staging), prior treatments received, reasons for not having surgery and/or conventional fractionated radiotherapy.
3. Charlson Co-morbidity Index (CCI) and ECOG performance Status
4. Quality of Life Questionnaire: Functional Assessment of Cancer Therapy-Head and Neck (FACT HN) [16].
5. Vulnerability and Frailty Assessments: Vulnerable Elders Survey 13 (VES13) [17] and Geriatric 8 (G8) [18].
6. No routine bloodwork is required for the current study
7. Diagnostic CT and/or MRI of the head and neck region with IV contrast as per the usual standard of care will be performed. Patients who have contra-indications to CT/MRI must have a clinically measurable skin lesion to be included.
8. For skin tumours, clinical measurements using calipers and clinical photographs will be recorded as per the usual standard of care

## Study treatment

### Stereotactic Body Radiation Therapy (SBRT)

#### Radiation dose

The dose prescribed in the study will be 45 Gy in 5 fractions, delivered once every 3–4 days, such that treatment is completed within 15 days (e.g. treatment given on Monday/Thursday/Mon/Thurs/Mon). Exceptions: treatment duration of up to 18 days will be allowed to account for cancer centre closures and unforeseen patient issues.

#### Simulation and planning

Radiation planning, simulation and treatment will adhere to the principles of SBRT described within the ASTRO-ACR and JCC practice guidelines [19]. Positioning and immobilization will be determined by the treating radiation oncologist and must allow the patient to be comfortable during the SBRT procedure. It is expected that all cases will utilize a thermoplastic immobilizing mask unless the patient is unable to tolerate the setup. Skin surface wires, clinical markings and clinical photographs will be used as per the usual standard of care for patients with tumours involving the skin. A planning CT scan will be performed for treatment planning using 1.5 mm image slice thickness. MR simulation will be performed when feasible, at the discretion of the treating radiation oncologist and fused with the CT sim images. IV contrast for CT and/or MR simulation will be used unless there are contraindications to contrast dye.

#### Target delineation and treatment of the primary tumour

The prescription dose GTV45 volume will be contoured on neck / soft tissue windows by the treating radiation oncologist to include all tumour regions and abnormal tissue on CT and MRI simulation images. No high dose CTV expansion will be used. A 5 mm expansion from GTV45 will be used to determine the high dose PTV45.

A microscopic dose CTV volume of 27 Gy/5 fractions will be contoured for the primary tumour. CTV27 will consist of the GTV with an expansion of 10 mm to encompass microscopic disease excluding air and anatomic boundaries such as bone and adjacent intact muscle planes. An additional 5 mm expansion will be used to determine the microscopic dose PTV27.

#### Target delineation and treatment of nodal disease

There will be no elective nodal radiotherapy for patients with stage N0 disease. Patients with regional nodal disease (biopsy proven or radiologically short axis ≥ 1 cm or with malignant features) will undergo treatment to encompass all involved nodes. A volume defined as “GTV45_nodal_” will be contoured by the radiation oncologist on neck/soft tissue windows to encompass all abnormal regional lymph nodes. No high dose CTV45_nodal_ volume will be used. A 5 mm PTV volumetric expansion from GTV45_nodal_ will be used to determine PTV45_nodal._

A microscopic dose CTV volume of 27 Gy will be contoured for nodal volumes. CTV27_nodal_ will consist of the nodal GTV with an expansion of 2 cm to encompass microscopic nodal disease excluding air and anatomic barriers such as lung, bone, muscle unless there is suspicion of direct invasion. A 5 mm expansion will be used to determine the PTV27_nodal_ contour.

#### Radiation treatment technique

Volumetric Modified Arc Therapy (VMAT), Intensity Modulated Radiotherapy (IMRT) or Cyberknife robotic radiosurgery are preferred treatment techniques for this study and will be determined by the treating radiation oncologist, in consultation with the medical physicist and radiation therapist.

Cone beam CT will be used for daily image guidance and matched to the primary tumour as well as organs at risk. Radiation treatment is to start within 14 days of the CT/MR simulation.

#### Radiation dose constraints (Additional file 2)

Radiation prescription to targets will be such that the following criteria are met:

1. GTV45 V100 > 95% and PTV45 V95 > 95%
2. PTV45 Dmax < 115% and mean dose < 105%
3. PTV45 V107 < 8%

#### Organs at risk – dose volume constraints (Additional file 2)

SBRT of the head and neck region is a relatively recent treatment approach. Therefore, radiation limits for the current study are based on a published survey of current HN SBRT practices at 15 international institutions as well as published experiences from other disease sites [12].

### Treatment verification

Verification of linear accelerator treatments will be performed daily using cone beam CT (CBCT) prior to each fraction of treatment. CBCT will be matched to the high dose GTV and adjacent soft tissue and bony structures. Patients treated with robotic radiosurgery will require real-time tumor tracking.

### Treatment review

Patients will be seen once weekly during or following treatment in review clinic to monitor any adverse events, as per the usual standard of care.

## Radiation therapy quality assurance

All cases on study will undergo a real-time review prior to the start of treatment by study radiation review committee, consisting of a radiation oncologist, medical physicist and radiation therapist (planner) to confirm that the plan meets the target delineation, target coverage and organ at risk constraints described in the protocol. In addition, all plans will be reviewed at standard head and neck disease site group weekly quality assurance rounds.

## Adjuvant systemic supportive therapy

### Permitted concomitant medication and therapy

Standard supportive treatments including patients` regular medications, antibiotics, analgesics, inhalers. No concurrent or adjuvant chemotherapy/immunotherapy is permitted. Palliative chemotherapy/immunotherapy is permitted three months after completion of the study treatment if indicated due to locoregional progression or at any time if the patient develops distant metastatic disease; currently the majority of patients in this study population do not receive palliative systemic therapy due to limited response rates, toxicity, co-morbidities and patient preference.

### Prohibited concomitant medication and therapy

Any non-standard systemic treatment as an adjuvant treatment.

### Supportive therapy

There is no planned additional supportive therapy during protocol treatment. However, therapy used to aid in subject comfort and symptoms during treatment (pain medications, inhalers) are permitted. Standard of care during head and neck radiotherapy typically includes skin moisturizer or topical antibiotics, sodium bicarbonate mouthwash or medicated mouthwashes, and analgesics as required.

## Evaluations during and after radiotherapy

### Assessment and evaluations during treatment: (Additional file 1).

1. Once per week during the treatment the patient will undergo the standard review clinic with the nursing staff and radiation oncologist to assess acute toxicity. This is the usual standard of care.
2. The research associate will administer and record acute toxicity, and quality of life questionnaires once during radiotherapy, at the time of the 4^th^ or 5^th^ treatment.

### Post treatment assessment and evaluations: (Additional file 1).

1. The patient will be seen at four assessments following completion of therapy at 6 weeks, and then at 3, 6, 12 months. Follow-up imaging with CT and/or MRI of the head and neck region will be performed 10–12 weeks after the last radiation treatment as per the usual standard of care. Toxicity, quality of life questionnaires, ECOG performance status assessment, will be administered at each follow-up visit and the tumour dimensions from clinical examination and/or any diagnostic imaging will be recorded. Any additional follow-up investigations/imaging will be done at the discretion of the oncology team. Regular follow-up after the 12 month visit will continue as per the usual standard of care. A chart review and data extraction will be performed by the clinical research associate to assess local control and progression free survival 2 years after completion of the treatment.

### Early permanent discontinuation of radiotherapy.

All definitive treatment discontinuation should be recorded by the Investigator, along with reasons for discontinuation.

#### Criteria for early permanent discontinuation of radiotherapy

Study treatment will be stopped prematurely and permanently if any of the following events occur or are diagnosed:

- Severe acute toxicity requiring hospitalization
- Significant change in clinical status due to comorbid illness such that the subject is unable to continue with protocol treatment.

### Follow-up after early permanent discontinuation of radiotherapy.

Subjects who meet the criteria for early permanent discontinuation of radiotherapy and do not receive further protocol treatment will continue to be followed for cancer recurrence. Efforts should be made to continue the follow-up schedule for survival unless the subject withdraws their consent from collection of data beyond the point of withdrawal from the protocol treatment.

## Cancer recurrence and other cancer events

At the time of first cancer recurrence, all subjects will be reassessed, undergo clinical examination, and the site of recurrence will be recorded as will treatment at time of recurrence. Re-staging investigations including chest x-ray, CBC, liver function tests, bone scan, and liver ultrasound or chest /abdominal CT will be done at the discretion of the most responsible physician and the oncology team. Treatment of recurrence is at the discretion of the treating oncologist and may include further radiotherapy, chemotherapy, targeted therapy, surgery, or supportive care. Subjects will continue to be followed for the primary outcome. Cancer recurrence and survival data pertaining to the secondary outcome (refer to Sect. 11) will be collected.

## Study outcomes

### Primary outcome

The primary outcome is tumour response rate (complete or partial response) as defined by RECIST 1.1 criteria [20]. Tumour response is the best overall response across all time points during the study period – up to 24 months after completion of SBRT.

### Secondary outcomes.

#### Toxicity

Acute (during and up to 3 months from the end of treatment) and late (after 3 months) adverse effects secondary to SBRT treatment will be graded according to the Common Terminology Criteria for Adverse Events (CTCAE v5.0) scale [21]. We expect less than thirty percent of patients will develop grade 3 or higher toxicity based on SBRT literature.

#### Local Control (LC)

Defined as the absence of local progression of disease of the target lesions during the study period. Operationally, this is the time from enrollment registration to progression of the treated lesion(s). Subjects without local recurrence will be censored at the earliest date of last follow-up or death. Determination of local recurrence will be based on RECIST criteria 1.1 (see Sect. 11.3 below) and assessed on MRI or CT scan imaging at 12 weeks and any additional imaging/clinical assessments performed during the study.

#### Quality of Life (QoL)

QoL will be assessed using FACT-HN questionnaire at baseline, once during treatment at the 4^th^ or 5^th^ fraction, at 6 weeks, and then at 3, 6, 12 months follow-up.

### Application of RECIST 1.1 for SBRT outcomes assessment

The RECIST 1.1 criteria were developed using data from cytotoxic drug trials. Radiotherapy is excluded in the majority of studies using RECIST and therefore must be clearly defined for use in the setting of this SBRT study. In this radiation study the ‘target lesions’ must be pathologically enlarged and measurable as per the RECIST 1.1 criteria: primary tumour ≥ 10 mm measured in the longest axis, and lymph nodes ≥ 15 mm in the shortest axis. CT imaging is the preferred method of measurement in all cases; exceptions will be allowed only if the patient is unable to have CT imaging of the target lesion and also has a superficial skin tumour that can be reproducibly measured using calipers. Lymph nodes ≥ 10 mm and < 15 mm are considered non-target, non-measurable lesions and will be documented but not considered in the radiation response assessment. A maximum of 4 target lesions will be measured at baseline: up to 2 primary tumour targets and the 2 largest target lymph nodes. A sum of diameters of the target lesions will be calculated based on the baseline CT scan and all subsequent CT scans to quantify response.

The baseline CT scan will be done no more than 4 weeks prior to the start of SBRT; CT simulation scan measurements will be used if the diagnostic scan is > 4 weeks prior to SBRT.

Complete response (CR) is defined as disappearance of the target lesion for primary tumours AND reduction of target lymph nodes to < 10 mm in the shortest axis for lymph node metastasis. Partial response (PR) is defined as at least 30% decrease in the sum of diameters of the target lesions [20, 22]. Local progression of disease (PD) in-field is defined as ≥ 20% increase in the sum of diameters of the target lesions measured at baseline or smallest sum of diameters measured in follow-up. Stable disease (SD) is defined as neither sufficient shrinkage from baseline or sufficient increase from smallest sum of diameters to qualify as CR, PR, or PD. New pathologically enlarged lesions outside of the target lesions and/or new distant metastases will be documented and defined as progression of disease outside the high dose radiation field – and will be further sub-classified as nodal or distant. Response assessment must be a minimum of 6 weeks from the start of SBRT to qualify as CR, PR or SD. All eligible patients will have follow-up imaging scheduled 10–12 weeks following the completion of treatment which will be used to assess tumour response. No additional confirmatory imaging is required on the study; if any additional diagnostic imaging is performed by the treating physicians then it will be included in the assessment of response / progression status. The best overall response across all time points will be used.

## Statistical considerations

### Sample size calculation and assumptions

Up to a total of 38 patients will participate in this single institution, single arm, phase 2 study. Based on prior research, the response rate for patients treated using the’0–7-21’ regimen was 82%, however, 6-month progression-free survival was only 39%. Given the expected relative minimal toxicity and ease of treatment using this regimen, a similar response rate of around 80% would be of considerable interest, while a response rate of 60% or lower would indicate that there would not be any interest in studying this regimen further. Hence, for sample size calculations, it will be assumed that H0: *p* = 60% versus HA: *p* = 80%, with α = 0.10 and β = 0.10. Using the Simon optimal two-stage design, 11 patients will be recruited in the first stage. If 6 or less patients have a response, the study will be stopped at the end of stage I, however, if 7 or more patients have a response the study will continue to stage II, in which an additional 27 patients (for a total of 38) will be recruited. At the end of stage II, if 26 or less patients have a response, then H0 will be accepted and the treatment regimen deemed not worthy of further study, however, if 27 or more patients have a response, the treatment regimen will be recommended for further study in phase III. The true α = 0.097 and β = 0.096 for this design, with a probability of termination at the end of stage I of 0.467.

### Statistical analysis

Tumour response and control will be reported based on all available clinical/imaging measurements and classified using the RECIST criteria. Adverse events will be summarized using the NCI-CTCAE. The number of patients with grade ≥ 2 toxicity will be reported. Any grade 4–5 toxicity or adverse event on the study will be reported. Baseline demographics, tumour characteristics and reason for no surgical management will be reported with descriptive statistics. The baseline Charlson Comorbidities Index, VES13 and G8 scores will be summarized to assess the study population for vulnerability/frailty. Quality of life and symptom scores will be assessed to identify the severity and duration of changes to these scores during and after SBRT. Exact confidence intervals will be constructed for outcomes of interest and statistical significance will be defined as a two-sided α ≤ 0.05 for all analyses other than the primary outcome. No interpolation will occur for any missing information.

All patients who receive at least one protocol defined treatment will be included in all analyses. Any patient registered to this study who does not receive protocol defined treatment will be described, including reasons for not receiving treatment, but will not be included in further analyses.

## Safety monitoring plan

Patients treated with HN SBRT on this study are anticipated to have significant frailty and co-morbidities that preclude surgery, conventional radiotherapy and systemic therapy. Studies involving a similar patient population at JCC and other centres reported median survival between 5–6 months and deem the treatment intent to be palliative [9-11]. Head and Neck cancer patients in Ontario are known to have a high rate of emergency room visits and hospitalizations [3]. Therefore, it is likely and expected that some patients may be admitted to hospital or die during the duration of the study; this may be caused by complications due to the tumour and associated treatments or for reasons unrelated to the cancer diagnosis.

A data safety monitoring committee (DSMC) consisting of at least two radiation oncologists who are not co-investigators in the study will review all grade 4 or 5 (severe) adverse events. Any severe adverse events will be classified as definitely related, possibly related or not related to the study intervention. Toxicity will be reviewed by the DSMC on a q6 monthly basis or more frequently if unexpected toxicity is observed. If grade 5 toxicity is observed in more than 10% of patients, the protocol will be amended to a lower radiation dose (40 Gy) for REB review, or the study may be closed.

## Ethical and regulatory standards

This clinical trial will be conducted in accordance with the recommendations guiding physicians in biomedical research involving human patients adopted by the 18^th^ World Medical Assembly, Helsinki, Finland 1964 and later revisions or the laws and regulations of the country, whichever provide the greater protection for the study participant.

This clinical trial will be conducted in compliance with the International Conference for Harmonisation guidelines for Good Clinical Practice and will adhere to national laws and regulations of the country in which the study is performed.

Personnel involved in conducting this clinical trial will be qualified by education, training and experience to perform their respective tasks.

### Informed consent

The Informed Consent Form (ICF) for obtaining the patient’s informed consent must be reviewed and approved by local Research Ethics Board (REB).

It is the responsibility of the Principal Investigator or a person designated and under the Investigator’s responsibility, to provide each potential study patient, prior to inclusion in the study, full and adequate verbal and written information regarding the objectives and procedures of the study and the possible risks involved. The patient must be informed about their right to withdraw from the study at any time. The patient must be allowed adequate time to make an informed decision.

Prior to a patient’s participation in the study, the ICF must be signed, name filled in and personally dated by the patient or by the patient’s legally acceptable representative, and by the person who conducted the informed consent discussion. A copy of the signed and dated written consent form document and any other written information should be provided to the patient.

Until the patient has been completely informed of the clinical trial, has freely consented to take part in the study and has signed and dated an ICF that has received documented approval by a licensed REB, no study related procedures can be performed.

### Research ethics board

During the clinical trial, any amendments or modification to the study protocol or ICF document must be submitted to and approved by the local REB. The REB should also be informed of any event likely to affect the safety of patients or the continued conduct of the study.

Annual re-approval is required for as long as the study is open to patient accrual, study participants are being followed and until the data collection and sponsor close-out is completed. The REB must be informed when the study is closed or has been suspended.

### Data storage and protection

The study data will be kept for a minimum of ten years after completion of the study. The local data will be destroyed by a secure, insured facility with tracking and documentation provided.

## Discussion

Overall it is recognized that elderly and/or medically frail patients with HNSCC are a vulnerable population that is underrepresented in clinical trials and require novel treatment approaches that can provide tumour control and symptom relief while limiting the burden of treatment [11, 23]. There is also a need for prospective clinical studies to better define the role of SBRT in unresected tumours of the head and neck region. Patients with non-metastatic disease are currently receiving a wide variety of radiation regimens associated which may be associated with significant toxicity and a high rate of locoregional progression. Based on experience from other disease sites including the lung and liver, SBRT has the potential to drastically decrease treatment times, decrease toxicity and improve locoregional control – all of which are highly relevant outcomes for head and neck cancer patients [24, 25].

More than 500 new patients per year are seen in consultation at the head and neck multi-disciplinary clinic at JCC. In addition, prior patients are seen for regular follow-up by the oncology team. We estimate that approximately 6 patients per month will meet eligibility criteria and 2 patients per month will decide to participate in the study. As such, we believe that we will be able to accrue the sample size required for this study.

This study may provide much needed prospective data on the use of SBRT in unresected HNSCC. For patients who have non-metastatic, unresectable tumours, SBRT may offer a shorter and more effective treatment than the current standard of care palliative regimens. If the study demonstrates that the treatment is feasible, safe and effective, then this may lead to a randomized study comparing conventional radiotherapy to SBRT for head and neck cancer in selected patients.

## Supplementary Information

**Additional file 1. **Summary of patient timeline and assessments on study.

**Additional file 2. **Organs at risk – radiation dose constraints.

## Data Availability

The datasets generated and/or analyzed during the current study are not publicly available but are available from the corresponding author on reasonable request.

## Abbreviations

ASTRO-ACR: American Society for Therapeutic Radiology and Oncology—American College or Radiology
CBCT: Cone Beam Computed Tomography
CCI: Charlson Co-morbidity Index
CT: Computed Tomography
CTCAE: Common Terminology Criteria for Adverse Events
CTV: Clinical Target Volume
ECOG: Eastern Co-operative Oncology Group
FACT HN: Functional Assessment of Cancer Therapy-Head and Neck Version
G8: Geriatric 8 (Frailty screening tool consisting of 8 questions)
GTV: Gross Tumour Volume
Gy: Gray (radiation dose units)
HN: Head and Neck
HNSCC: Head and neck squamous cell carcinoma
ICF: Informed Consent Form
IMRT: Intensity Modulated Radiation Therapy
IV: Intra-venous
JCC: Juravinski Cancer Centre
MRI: Magnetic Resonance Imaging
PTV: Planning Target Volume
QoL: Quality of Life
RCT: Randomized Control Trial
REB: Research Ethics Board
RECIST: Response Evaluation Criteria in Solid Tumors
SBRT: Stereotactic Body Radiotherapy
SCC: Squamous Cell Carcinoma
VES13: Vulnerable Elders Survey 13 (screening tool for risk of health deterioration)
VMAT: Volume Modulated Arc Therapy
XRT: Radiotherapy
