# Supplementary material for: A phase 2 study of stereotactic body radiation therapy for squamous cell carcinoma of the head and neck (SHINE): a single arm clinical trial protocol
Source: BMC Cancer. 2023 Apr 26;23:379. doi: 10.1186/s12885-023-10807-4 (PMC10131380; doi:10.1186/s12885-023-10807-4)
Supplement: Supplementary file 1 — Additional file 1. Summary of patient timeline and assessments on study. [file 12885_2023_10807_MOESM1_ESM.docx]

# Additional file 1: Summary of Patient Timeline and Assessments on Study

| Assessment | Pre-SBRT | During SBRT (4^th^ or 5^th^ treatment) | 6 weeks post- SBRT | 3 months post-SBRT | 6 and 12 months  post-SBRT | 24 months post-SBRT |
| --- | --- | --- | --- | --- | --- | --- |
| History and Physical Exam (standard) | X | X | X | X | X | X |
| Baseline Questionnaires (G8, VES, Charlson CI) | X |  |  |  |  |  |
| Tumour measurement* (RECIST) | X |  |  | X | X  (* if applicable) |  |
| QOL Questionnaire  (FACT HN) | X | X | X | X | X |  |
| Toxicity  (CTCAE) | X | X | X | X | X |  |
| Chart Review for Disease Status |  |  |  |  |  | X |

*****Tumour measurement for RECIST criteria is required at baseline and 12 weeks post-SBRT to assess tumour response. Any additional imaging will be done at the clinical discretion of the treating physician(s) and will be included in the assessment of best overall response and progression of disease.

After study completion clinical follow-up will continue with the treating physician(s) as per the usual standard of care.
