# Supplementary material for: A phase 2 study of stereotactic body radiation therapy for squamous cell carcinoma of the head and neck (SHINE): a single arm clinical trial protocol
Source: BMC Cancer. 2023 Apr 26;23:379. doi: 10.1186/s12885-023-10807-4 (PMC10131380; doi:10.1186/s12885-023-10807-4)
Supplement: Supplementary file 2 — Additional file 2. Organs at risk – radiation dose constraints. [file 12885_2023_10807_MOESM2_ESM.docx]

# Additional file 2: Organs at Risk – Radiation Dose Constraints

| Organ at Risk | Volume constraint | Maximum point dose | Endpoint toxicity |
| --- | --- | --- | --- |
| Spinal Cord |  | 28 Gy | Myelitis |
| Brain |  | 30 Gy | Symptomatic necrosis |
| Brainstem |  | 25 Gy | Symptomatic necrosis |
| Brachial Plexus |  | 32 Gy | Neuropathy |
| Carotid Artery |  | 45 Gy | Carotid blowout syndrome /stroke |
| Cochlea |  | 25 Gy | Hearing loss |
| Esophagus |  | 27.5 Gy | Stenosis |
| Larynx (uninvolved) | Mean dose <25 Gy |  | Laryngitis, stricture |
| Mandible (uninvolved by tumour) |  | 30Gy, up to 40Gy if overlaps with PTV45 | Osteoradionecrosis |
| Parotid (uninvolved) | Mean dose <25 Gy |  | Xerostomia |
| Pharyngeal constrictors | Mean dose <25 Gy |  | Dysphagia |
| Optic nerves / chiasm | V20 < 0.2cc | 25 Gy | Optic neuropathy, blindness |
| Oral cavity (uninvolved) | Mean dose <25 Gy |  | Xerostomia, mucositis |
| Skin (excluding PTV) | V103 < 0.1cc | 105% of SBRT dose | Ulceration, fibrosis |

PRV margin of 3mm will be used for the brainstem, optic nerves and chiasm. The spinal canal contour will be used as a PRV estimate for the spinal cord

If dose constraints for the brain, brainstem, spinal cord, carotid artery and optic structures cannot be met then the prescription and/or coverage will be lowered in 2.5 Gy decrements. If the constraints still cannot be met using a dose of 35 Gy / 5 fractions, then the patient will be removed from the study such that other doses may be considered at the discretion of the treating oncologist.
